# A reduced graphene oxide-β-cyclodextrin nanocomposite-based electrode for electrochemical detection of curcumin†

**DOI:** 10.1039/d0ra10701h

**Published:** 2021-02-18

**Authors:** Behzad Mirzaei, Ali Zarrabi, Abdollah Noorbakhsh, Abbas Amini, Pooyan Makvandi

**Affiliations:** Department of Biotechnology, Faculty of Biological Science and Technology, University of Isfahan Isfahan 81746-73441 Iran alizarrabi@sabanciuniv.edu; Sabanci University, Nanotechnology Research and Application Center (SUNUM) Tuzla 34956 Istanbul Turkey; Department of Nanotechnology Engineering, Faculty of Chemistry, University of Isfahan Isfahan 81746-73441 Iran; Centre for Infrastructure Engineering, Western Sydney University Penrith 2751 NSW Australia; Department of Mechanical Engineering, Australian College of Kuwait Mishref Kuwait; Chemistry Department, Faculty of Science, Shahid Chamran University of Ahvaz Ahvaz 6153753843 Iran Pooyanmakvandi@gmail.com

## Abstract

Curcumin is a polyphenolic compound with anti-oxidative and anti-cancer properties that is obtained from turmeric plants. Several studies have demonstrated that cancer cells are not killed unless they are exposed to 5–50 mM of curcumin. Consequently, it is vital to control the concentration of curcumin in cancer therapy. In this study, a sensitive electrochemical sensor was fabricated based on a beta-cyclodextrin–reduced graphene oxide (β-CD–rGO) nanocomposite for measuring curcumin concentration. The effects of experimental factors were investigated and the optimum parametric conditions were determined using the Taguchi optimization method. The β-CD–rGO modified electrode exhibited good electrochemical properties for curcumin detection. The results of differential pulse voltammetry experiments unveiled that the sensor shows a linear response to curcumin concentration over the range of 0.05–10 mM with a detection limit of 33 nM and sensitivity of 4.813 μA μM^−1^. The fabricated sensor exhibited selectivity in the presence of other electroactive species, *e.g.*, propranolol, clomipramine and clonazepam.

## Abstract 2

In this study, a sensitive electrochemical sensor was fabricated based on a beta-cyclodextrin–reduced graphene oxide (β-CD–rGO) nanocomposite for measuring curcumin concentration.

## Graphical abstract

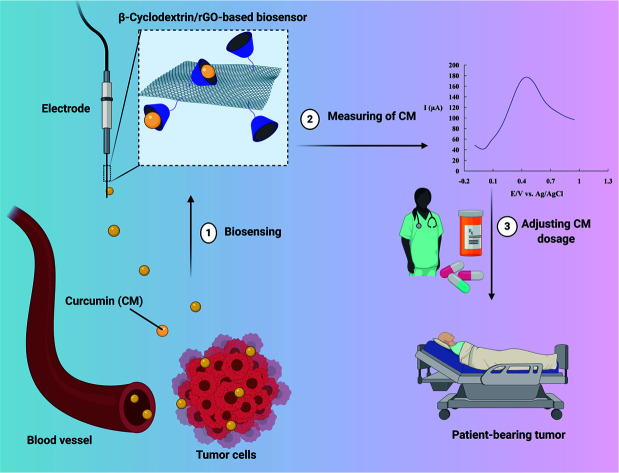

## 1 Introduction

Curcumin (CM) (1,7-bis(4-hydroxy-3-methoxyphenyl)-1,6-heptadiene-3,5dione) is a polyphenolic compound derived from turmeric, the rhizome of the *Curcuma longa* plant.^1^ This compound has anti-inflammatory, anticoagulant,^2^ antitumor^3,4^ and anti HIV^5^ properties.

CM suppresses cancer cell proliferation through the inhibition of inducible nuclear factor kappa B (NF-κB). The anti-inflammatory effect of CM is a consequence of the reduction of NF-κB, cyclooxygenase 2 (COX2) and tumour necrosis factor-α (TNF-α).^6,7^*In vitro* studies have shown that the cancer cells are not killed unless they are exposed to 5–50 μM of CM.^8,9^ There are several methods for CM detection, such as HPLC,^10–12^ UV fluorescence^13^ and electrochemical methods.^14–16^ Electrochemical sensors possess high sensitivity, portability, rapid measurement, simplicity and need a small quantity of sample.^17,18^ Commercial applications confirm the attractive advantages of these biosensors.^19,20^

In recent years, carbon nanomaterials and nanostructures have received significant attention as electrochemical sensors and biosensors due to their biocompatibility, high surface area, good electrical properties and chemical stability.^18,21,22^ Graphene-based nanomaterials with large surface area, excellent electron transportation and high thermal conductivity have shown great potentials for electrochemical biosensors.^23,24^ In this regard, graphene-based electrochemical sensors have been used for detecting glucose,^25^ dopamine,^26^ hemoglobin^27^ and heavy metal ions.^28^ Based on the unique properties of graphene (high surface area and superconductivity) and β-CD (supramolecular recognition due to their capability of forming inclusion complexes with many hydrophobic guest molecules^29–32^), the integration of graphene and β-CD can introduce a new nanocomposite which extends individual properties of both materials. CM, as a hydrophobic drug, can form inclusion complexes with β-CD molecules.^33,34^

In the present study, beta-cyclodextrin–reduced graphene oxide (β-CD–rGO) nanocomposite was introduced for the first time to build up a highly sensitive electrochemical sensor for quantifying CM. The nanocomposite was characterized using Fourier transform infrared spectroscopy, high-resolution transmission electron microscopy, electrochemical impedance spectroscopy, cyclic voltammetry and differential pulse voltammetry. Finally, the effects of various experimental parameters on the performance of the fabricated sensor were investigated through two different methods (*e.g.*, Taguchi and individual common optimization).

## 2 Material and methods

### 2.1 Material and reagents

CM, β-CD and graphite powder were purchased from Sigma Aldrich, Germany. Hydrazine solution, NaOH, HCl, dimethyl sulfoxide (DMSO), H_2_O_2_, H_2_SO_4_, H_3_PO_4_, K_2_HPO_4_, KH_2_PO_4_ and KMnO_4_ were obtained from Merck, Germany. Glassy carbon (GC) electrode was provided by Azar Electrode, Tabriz, Iran. All chemicals were of analytical grade and used without further purification. Hydrogen chloride (HCl) and sodium hydroxide (NaOH) were used for pH adjustment. Double distilled water was used throughout the work. All electrochemical experiments were carried out at room temperature 25 ± 0.1 °C.

### 2.2 Instrument and measurement methods

Fourier transform infrared (FTIR) spectroscopic measurements were carried out using a 6300 JASCO FTIR Spectrometer (Japan). All electrochemical measurements were carried out at room temperature using potentiostat/galvanostat Autolab PGSTAT (Eco Chemie, Utrecht, Netherlands; driven with NOVA software). These measurements were carried out with a conventional three-electrode system consisting of modified/unmodified GC electrode as a working electrode, a platinum wire as an auxiliary electrode, and an Ag/AgCl (3 M KCl) electrode as a reference electrode. Electrochemical Impedance Spectroscopy (EIS) was run in a 0.1 M KCl solution containing 5 mM K_3_[Fe(CN)_6_]/K_4_[Fe(CN)_6_] (1 : 1). EIS measurements were recorded under an oscillation potential of 5 mV over a frequency range of 10–0.1 Hz. Differential Pulse Voltammetry (DPV) was performed in 0.1 M phosphate buffer solution (PBS) (pH 7.4) with an amplitude of 25 mV and a pulse width of 0.05 s. To record DPV plots of CM, 1 mM CM was dissolved in DMSO, and the modified electrode was immersed in it for 30 minutes, then carefully washed with distilled water. Finally, DPV was performed at the potential range of 0.1–0.9 V, and current was plotted as a function of potential. High-resolution transmission electron microscopy (HRTEM) images were obtained using a JEM-2100F machine, Japan, operating at an accelerating voltage of 200 kV.

### 2.3 Synthesis of graphene oxide

Graphene oxide (GO) nanosheets were synthesized from graphite flakes by a modified Hummer's method.^35^ Typically, a mixture of H_2_SO_4_/H_3_PO_4_ (360/40 mL) was added to a mixture of graphite powder (3 g) and KMnO_4_ (1.8 g). The reaction mixture was warmed up to 50 °C and stirred for 12 h. The solution was cooled down to room temperature, and then, 200 mL of ice and 2.6 mL hydrogen peroxide (30%) were added to the mixture. The mixture was centrifuged, and supernatant was decanted away. The remaining solid material after multiple washing process (with ether, 30% HCl, ethanol and water) was vacuum-dried for 24 h to obtain GO powder.

### 2.4 Synthesis of β-CD–rGO nanocomposite

To fabricate β-CD–rGO nanocomposite, 10 mg GO and 20 mg β-CD were dispersed in deionized water (20 mL). Then, the solution was mixed with 300 μL ammonia solution and 20 μL hydrazine solution. After continuous stirring for a few minutes, the homogenous solution was placed in water bath (60 °C) for 4 h; the stable black dispersion was obtained. The dispersion was filtered with a nylon membrane (0.22 μm) to obtain β-CD–rGO nanocomposite.^36,37^

### 2.5 Preparation of modified electrodes

For pre-treatment of β-CD–rGO, 100 mg of β-CD–rGO powder was dispersed in 10 mL water and sonicated for 20 minutes to obtain a homogenous solution of β-CD–rGO. A bare GC electrode was polished with 0.3 μm and 0.05 μm alumina powder, and carefully washed with ethanol and distilled water. The modified glassy carbon electrode β-CD–rGO (β-CD–rGO/GC) was constructed by drop coating of 2 μL β-CD–rGO solution (2 mg mL^−1^) onto the surface of GC electrode and dried in air.

### 2.6 Experimental design

Taguchi method^38^ is a powerful tool for designing experiments and analyzing the effect of control factors. We defined four important factors for the optimization of CM percentage, as follow: incubation time (minutes), β-CD/GO mass ratio, β-CD–rGO concentration (g mL^−1^), and electrolyte pH (Table SI-1, ESI†). The level of factors was selected by varying them in a range according to the experimental optimization configuration. The present design includes four control factors, three factors with three levels, and one with two levels, which total the experiments to 18.

## 3 Results and discussion

### 3.1 Characterization of β-CD–rGO nanocomposite

**Fig. 1:**
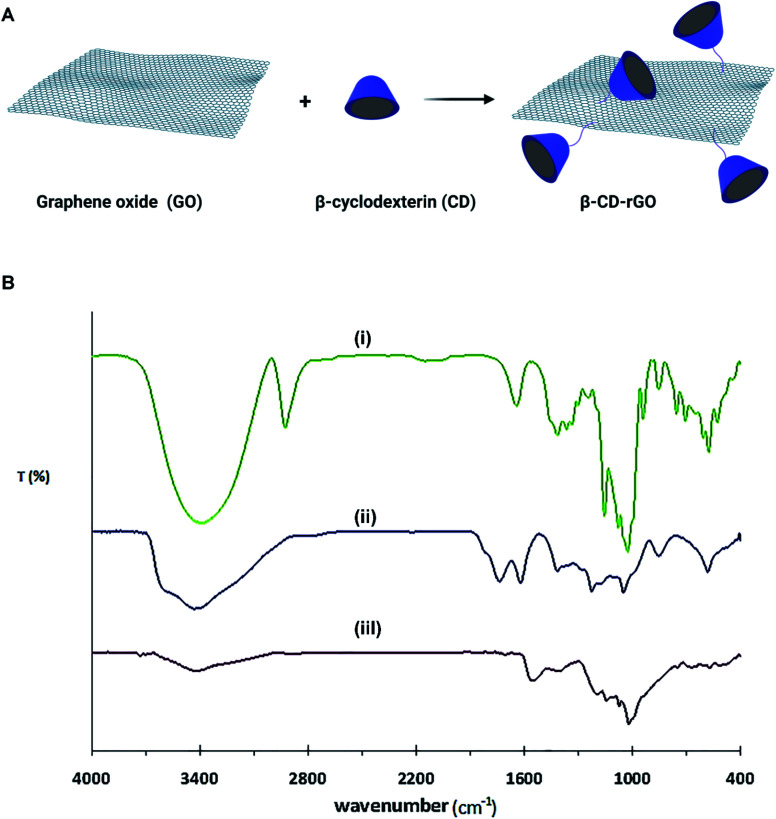
(A) Schematic illustration on the synthesis process of β-CD–rGO nanocomposite. (B) FTIR spectra of β-CD (i), GO (ii) and β-CD–rGO (iii).

Fig. 1A describes the formation process of β-CD–rGO nanocomposite. Aqueous suspension of β-CD–rGO nanocomposite was obtained after the sonication of rGO mixture (1 mg mL^−1^) and β-CD solution (1 mM). Hydrophobic noncovalent host–guest interactions between β-CD and rGO are responsible for the formation of β-CD–rGO composite.^39–41^

Fourier-transform infrared spectroscopy (FTIR) spectra of β-CD, GO and β-CD–rGO nanocomposite are shown in Fig. 1B. FTIR spectra of GO confirms the presence of oxygen containing groups: C–O vibration at 1051 cm^−1^, C–O–C vibration at 1178 cm^−1^, C–OH vibration at 1404 cm^−1^, C=O in carboxylic groups at 1738 cm^−1^, and OH stretching vibration at 3429 cm^−1^.^36,42^

The major bands for β-CD molecules are located at 707, 756, 857 and 943 cm^−1^. These peaks reveal the presence of ring vibration (characteristic peaks) for β-CD. The major absorption peak is located at 3396 cm^−1^ that is assigned to OH stretching vibration.^36,43^ When β-CD molecules were introduced to rGO surface, β-CD–rGO nanocomposite was formed. As shown in the FTIR spectra of β-CD–rGO nanocomposite, several characteristic peaks of β-CD molecules are observed which indicates that β-CD molecules attached to the surface of GO. It is also seen that the OH stretching vibration exhibited typical red-shift when hydrogen bonding was formed.^44^

When β-CD molecules were assembled on the surface of GO, the OH stretching vibration (3427 cm^−1^) in β-CD–rGO exhibited red-shift relative to OH stretching vibration in β-CD (3396 cm^−1^). From the FTIR data, both GO and β-CD had multiple OH-groups, so, when the β-CD molecules were introduced to the GO solution, hydrogen bonding was formed; this finding is in good accordance with previous results.^36,45^

**Fig. 2:**
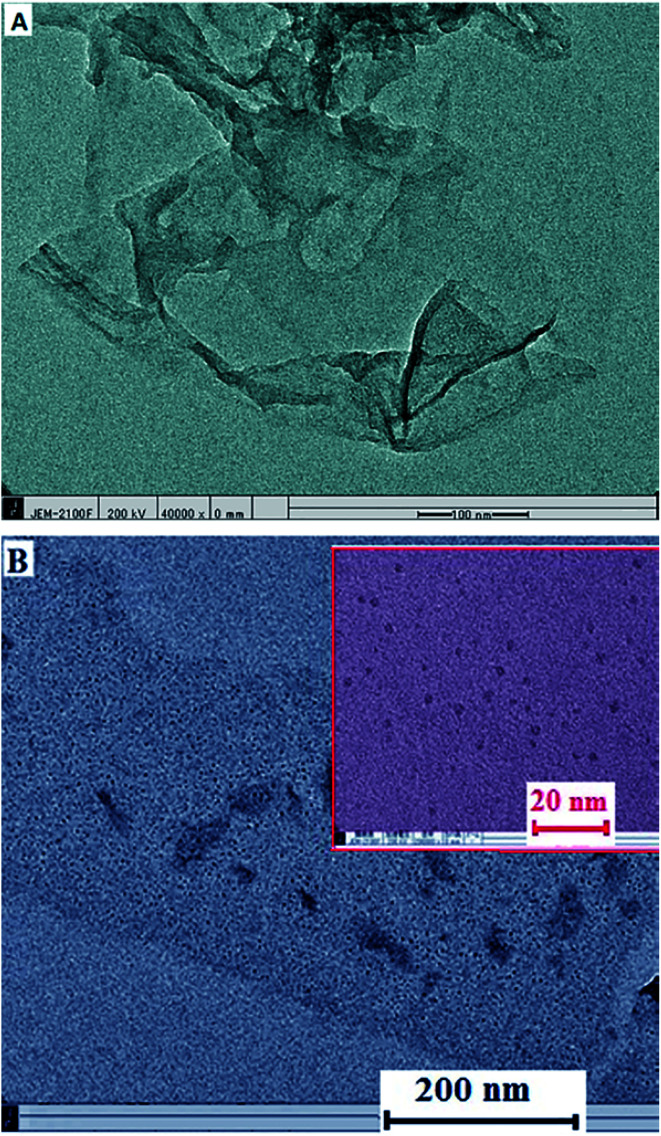
HRTEM images of GO (A), and β-CD–rGO nanocomposite (B). Inset shows the β-CD–rGO nanocomposite at higher magnification.

HRTEM images determined the GO sheets with flake-like shape (Fig. 2A), which is the evidence of successful GO sheets synthesis.^46,47^ The HRTEM image of β-CD–rGO (Fig. 2B) indicates the successful linkage of β-CD molecules with the surface of rGO. It also displays a homogenous surface with uniform distribution of β-CD on the surface of rGO.

### 3.2 Surface analysis of modified electrode

**Fig. 3:**
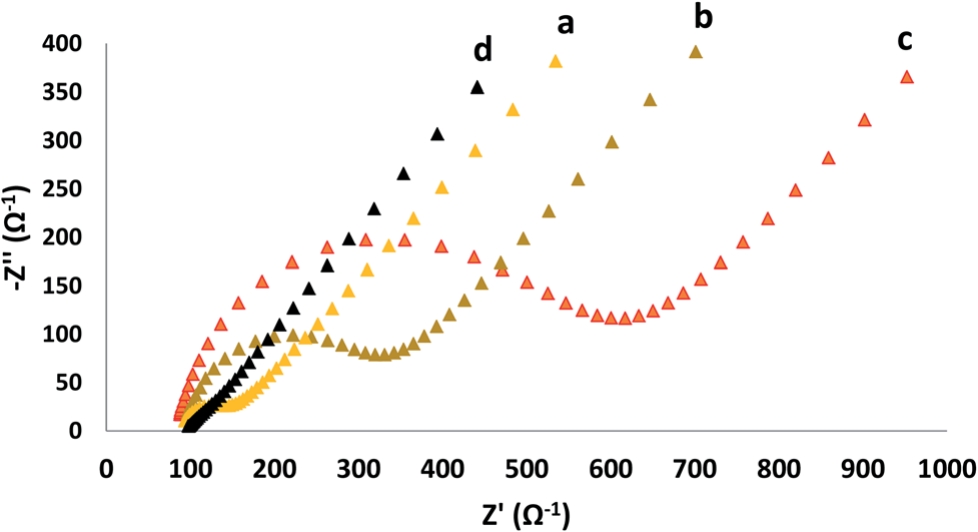
Nyquist plots of GC (a), GC/GO (b), GC/β-CD (c) and GC/β-CD–rGO (d) electrodes. EIS experiments were carried out in 0.1 M KCl solution containing 5 mM K_3_Fe(CN)_6_/K_4_Fe(CN)_6_ under a constant potential of 0.22 V and a frequency range of 10–0.1 Hz.

The semicircle portion of Nyquist diagram addresses the electron charge resistance; the charge transfer resistance can be directly measured *via* the semicircle diameter. This method is a useful way to study the surface properties of modified electrode. Fig. 3 shows the Nyquist plots of GC electrode, GO modified GC electrode (GC/GO), β-CD modified GC electrode (GC/β-CD) and β-CD–rGO modified GC electrode (GC/β-CD–rGO) in the presence of 10 mM K_3_Fe(CN)_6_/K_4_Fe(CN)_6_ and 0.3 M KCl. For the GO modified GC electrode and β-CD modified GC electrode, the semicircle portion of corresponding Nyquist diagrams dramatically increased in comparison with bare GC electrode as the semiconducting properties of GO and β-CD modified GC electrode increased. Also, the electrostatic repulsion between negative oxygen groups on the surface of GO and β-CD molecules with negatively charged electrochemical probe (K_3_Fe(CN)_6_/K_4_Fe(CN)_6_) increased the charge transfer resistance. When the GC electrode was modified with the β-CD–rGO nanocomposite layer, the semicircle portion dramatically decreased compared to bare GC electrode; this phenomenon is attributed to good electronic properties of β-CD–rGO nanocomposite. Thus, β-CD–rGO nanocomposite can greatly increase the electron transfer kinetics and provide a suitable environment for electron transfer.

### 3.3 Electrochemical behavior of curcumin

**Fig. 4:**
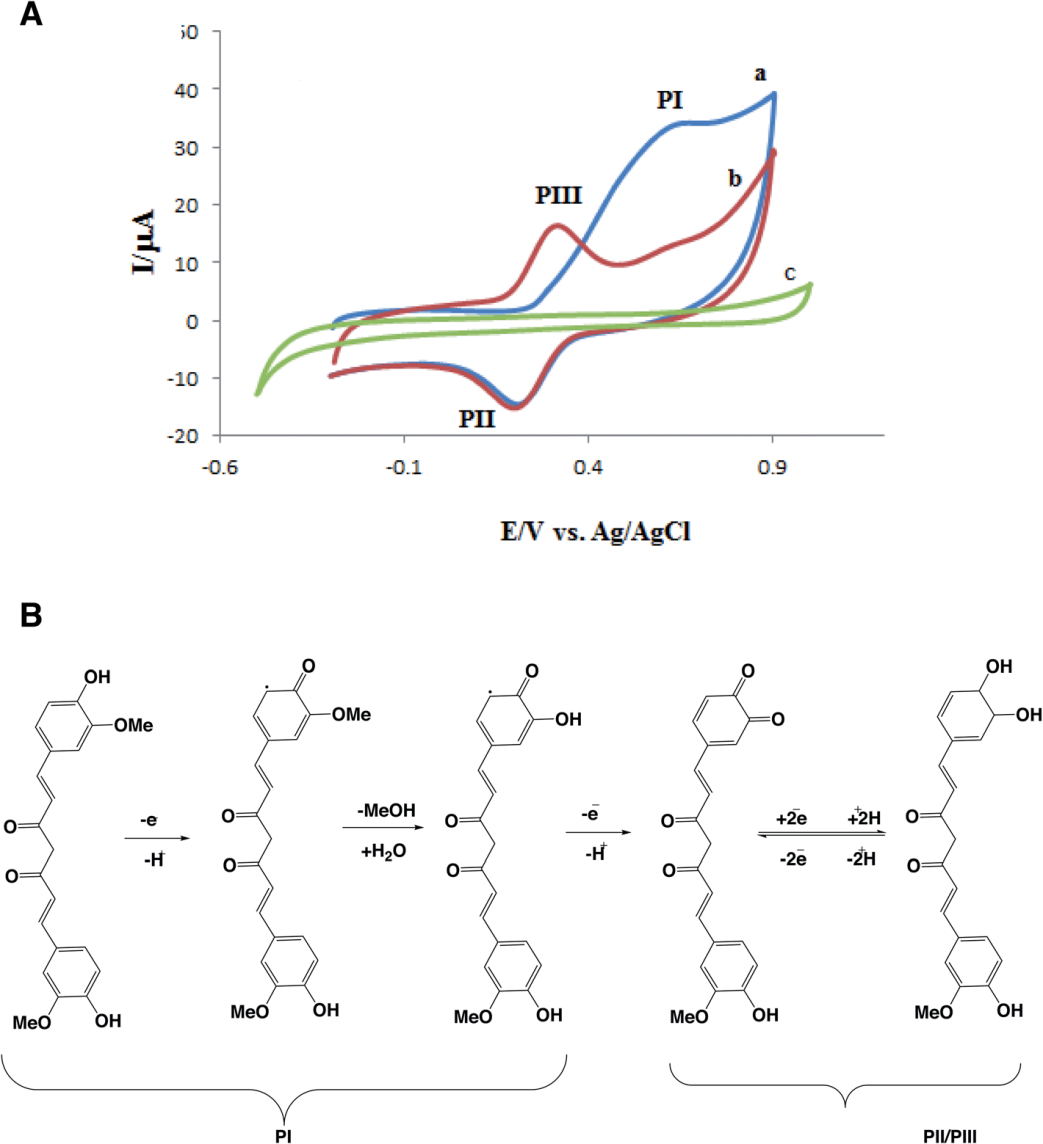
(A) Cyclic voltammograms of GC/β-CD–rGO electrode in the presence (a and b for two successive cycle) and absence (c) of 1 mM CM in 0.1 M phosphate buffer solution pH 7.4 at the scan rate of 50 mV s^−1^; (B) electrochemical oxidation of CM.

CM has functional phenolic hydroxyl groups and methoxy groups which can be oxidized at the surface of electrode. The hydroxyl groups, present at the benzene rings, can easily undergo an oxidation process.^48,49^ In fact, the electrochemical oxidation of CM has two-steps.^50^Fig. 4A shows one cyclic voltammogram of GC/β-CD–rGO electrode in the absence of CM and two successive cycles in the presence of 1 mM CM, both in the phosphate buffer solution (pH 7.4). An irreversible oxidation peak I and a pair of reduction/oxidation peak (II/III) are observed in the presence of CM. In the first cycle, CM exhibits an oxidation peak I and a reduction peak II with *E*_paI_ = 0.7 V and *E*_pcII_ = 0.21 V. In the second cycle, a new anodic peak III appears with *E*_paIII_ = 0.31 V while the oxidation peak I is disappeared. According to others' findings, the oxidation peak I is an irreversible step and its active group comes from the product of irreversible reaction.^51,52^

**Fig. 5:**
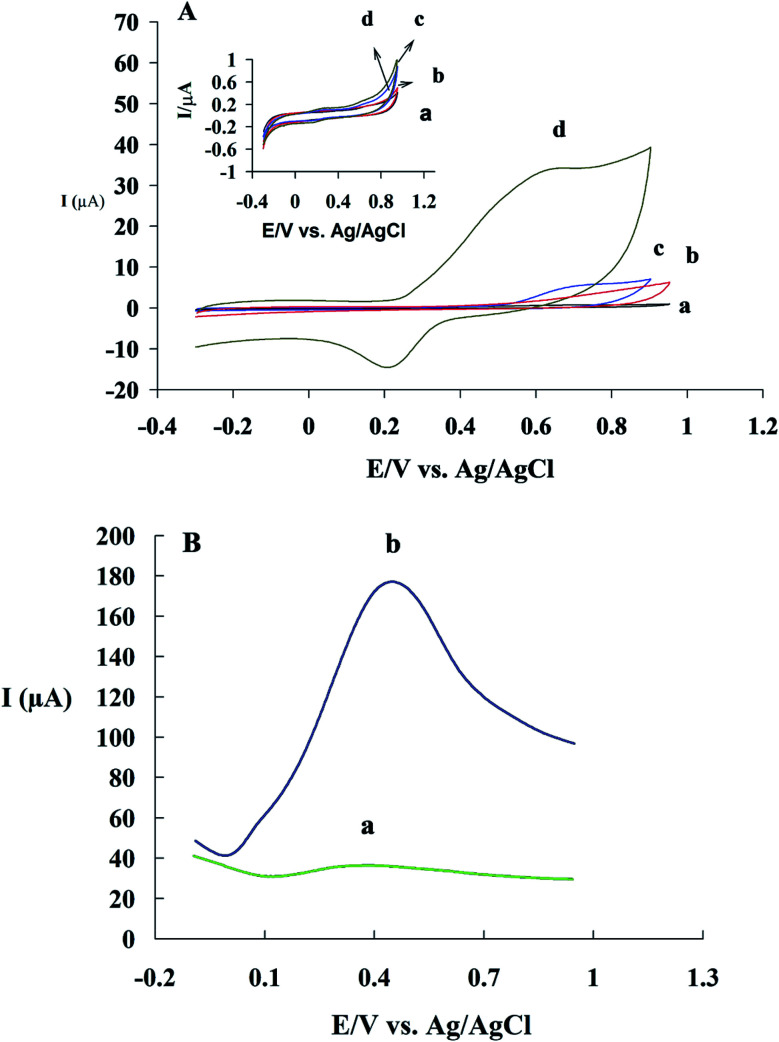
(A) Cyclic voltammograms of bare GC (a), GC/β-CD (b), GC/GO (c) and GC/β-CD–rGO (d) electrodes after the incubation with 1 mM CM in 0.1 mM phosphate buffer solution (pH 7), free of CM, at the scan rate of 50 mV s^−1^. Inset A shows the cyclic voltammograms of these electrodes without incubation under the same conditions. (B) Differential pulse voltammograms of GC/β-CD–rGO electrode before (a) and after (b) incubation with 1 mM CM in 0.1 mM phosphate buffer solution (pH 7), free of CM, at the scan rate of 10 mV s^−1^.

The electrochemical behaviors of GC (voltammogram a), GC/β-CD (b), GC/GO (c) and GC/β-CD–rGO (d) electrodes are compared in Fig. 5A, after incubation with 1 mM CM in 0.1 mM phosphate buffer solution, free of CM, at the scan rate of 50 mV s^−1^. Inset Fig. 5A shows cyclic voltammograms of these electrodes in 0.1 mM phosphate buffer solution, free of CM, at the similar scan rate. No oxidation current was observed at the voltammograms of electrodes before incubation with CM. After incubation with CM, an obvious CM oxidation peak was observed at the surface of GC/β-CD–rGO electrode. The oxidation peak of CM appeared at 0.7 V *vs.* Ag/AgCl electrode which was attributed to the phenolic groups of CM. This was an irreversible oxidation process, in good accordance with previous reports.^53,54^ A very small oxidation peak at the surface of GC/GO and GC/β-CD was also observed. These results demonstrated that CM intensely accumulated at the surface of GC/β-CD–rGO during the incubation, and, β-CD–rGO modified electrode significantly increased the sensitivity of electrode for CM sensing/measuring.

The electrochemical oxidation properties of GC/β-CD–rGO electrodes were investigated using DPV. This technique is an effective method for measuring the electroactive species. Fig. 5B shows differential pulse voltammograms of GC/β-CD–rGO electrode before (voltammograms a) and after accumulation with 1 mM CM (b) in phosphate buffer electrolyte solution (pH 7), free of CM. After the incubation with CM, an obvious electrochemical oxidation peak was observed which was originated from the accumulation of CM at the surface of electrode.

### 3.4 Scan rate study

**Fig. 6:**
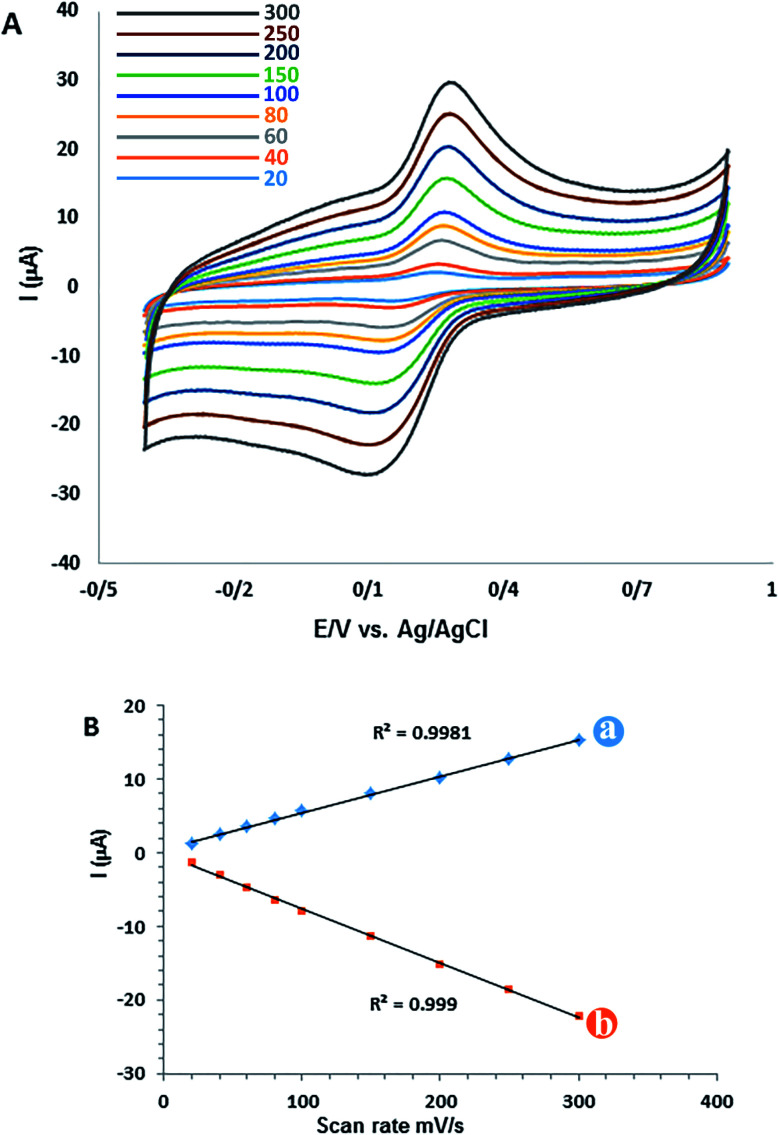
(A) Cyclic voltammograms of CM *versus* scan rate obtained from 50 μM CM for the GC/β-CD–rGO after two cycles at different scan rates (from inner to outer): 20, 40, 60, 80, 100, 150, 200, 250 and 300 mV s^−1^ in 0.1 mM phosphate buffer solution (pH 7). (B) Lines a and b are the plots of peak currents *versus* scan rate.

The scan rate study is an important stage in characterizing the electrochemical behaviour of CM for the GC/β-CD–rGO electrode. The effect of potential scan rate (*ν*) on both cathodic and anodic peak currents was investigated by cyclic voltammetry at different scan rates. Fig. 6A displays the cyclic voltammograms of 50 μM CM for GC/β-CD–rGO electrode at the scan rate ranged from 20 to 300 mV s^−1^. Both cathodic and anodic peaks are linearly proportional with the scan rate. The linear regression equations are *i*_pa_ (μA) = 0.40491*ν* (mV s^−1^) + 0.595 (μA) (*R*^2^ = 0.9981), and *i*_pc_ (μA) = −0.07365*ν* (mV s^−1^) + 0.2846 (μA) (*R*^2^ = 0.999), confirming the reaction as a surface-confined process.^55^

### 3.5 Optimization of experimental conditions for detecting curcumin

#### 3.5.1 Effect of incubation time

**Fig. 7:**
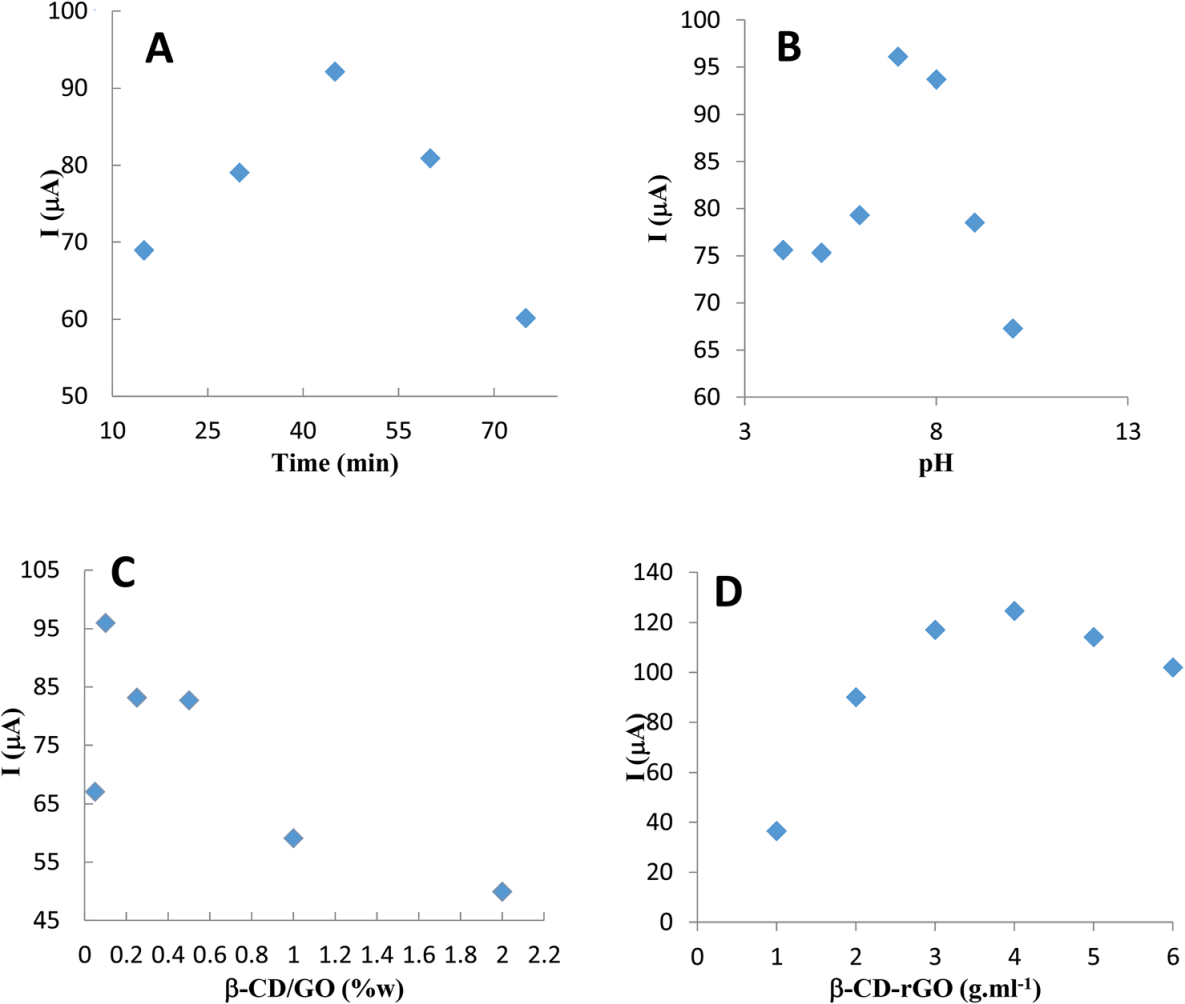
Plots of GC/β-CD–rGO electrode response from DPV at the scan rate of 10 mV s^−1^, after incubation with 1 mM CM, in 0.1 M phosphate buffer solution, free of CM, *versus*: (A) incubation time in phosphate buffer solution with pH 7, (B) pH of electrolyte solution in incubation time of 45 minutes, (C) mass ratio of β-CD/GO in phosphate buffer solution with pH 7 and incubation time of 45 minutes, (D) different concentrations of β-CD–rGO in phosphate buffer solution with pH 7 and incubation time of 45 minutes.

The effect of CM incubation time for the modified electrode was investigated *via* the oxidation current peak of 1 mM CM. For this purpose, the β-CD–rGO modified GC electrode was immersed in the CM solution for 15, 30, 45, 60 and 75 minutes, and the corresponding differential pulse voltammograms were recorded in the phosphate buffer electrolyte solutions (pH 7), free of CM. As shown in Fig. 7A, the maximum oxidation peak belongs to the 45 minutes incubation time, therefore, 45 minute time was selected as the optimum incubation time for the CM characterization.

#### 3.5.2 Effect of pH

The effect of pH on the electrochemical behavior of CM in the GC electrode modified with β-CD–rGO nanocomposite, was investigated after incubation with 1 mM CM in phosphate buffer electrolyte solutions, free of CM, with the pH range 4.0–10 using DPV (Fig. 7B). For all pH levels, an obvious electrochemical signal was observed. The oxidation peak potential of CM shifted positively when the pH increased; this indicated that the electrochemical oxidation of CM was proton transfer dependant. The maximum oxidation current for CM was obtained at pH 7.

#### 3.5.3 Characterizing of optimized mass ratio β-CD/GO

The effect of mass ratio β-CD/GO on the electrochemical oxidation of CM was investigated in the synthesis process of β-CD–rGO nanocomposite. β-CD–rGO nanocomposite was prepared with different mass ratios of β-CD/GO (0.05/1, 0.1/1, 0.25/1, 0.5/1, 1/1 and 1/2), and their corresponding β-CD–rGO modified electrodes. After incubation with 1 mM CM, the electrochemical behavior of these electrodes was examined in phosphate buffer electrolyte solution (pH 7), free of CM. As shown in Fig. 7C, the oxidation peak current of CM decreased with an increase of β-CD/GO mass ratio; this was attributed to non-conductive property of β-CD molecules. When β-CD/GO ratio was in the range of 0.1/1, the oxidation peak current maximized. At the lower β-CD/GO mass ratio, the oxidation current of CM decreased, due to the low concentration of β-CD which acted as a receptor for trapping CM. Thus, 0.1/1 was selected as the optimum state for sensor fabrication.

#### 3.5.4 Effect of β-CD–rGO quantity on surface modification and electrode performance

Modifying the amount of β-CD–rGO in the electrode surface can change the β-CD–rGO thickness and function of the electrode surface. To cover this, DPV of electrodes after incubation with CM were recorded for GC electrode modified for different amounts of 1, 2, 3, 4, 5 and 6 mg mL^−1^ β-CD–rGO suspension. The oxidation peak current increased by increasing the concentration of composite from 1 to 4 mg mL^−1^ (Fig. 7D). For the concentrations up to 4 mg mL^−1^, the oxidation peak current decreased, because by increasing the concentration of composite at the surface of electrode, the material was wasted. Therefore, 4 mg mL^−1^ was selected as the optimum concentration for fabricating the modified electrode.

### 3.6 Taguchi design results

**Fig. 8:**
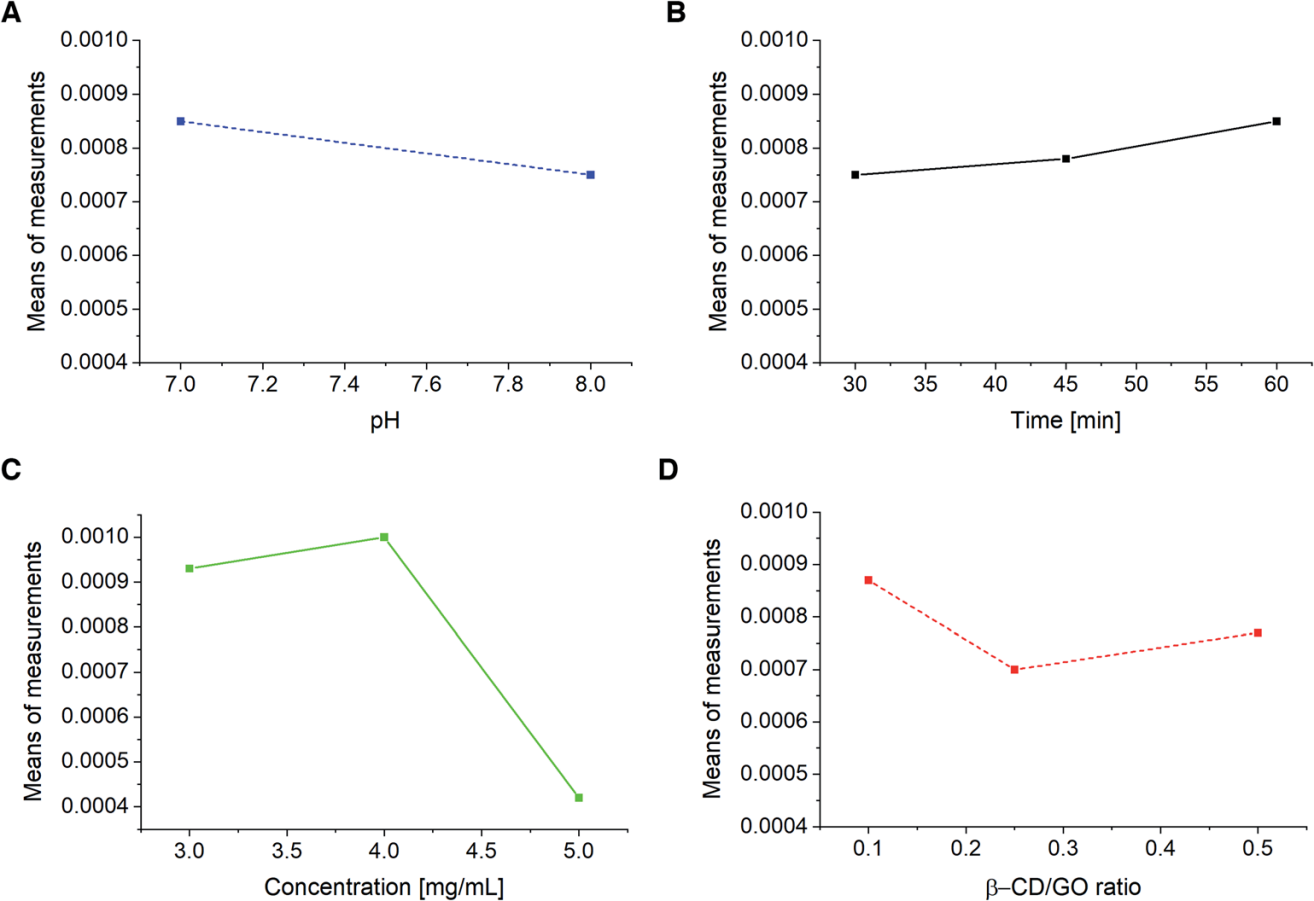
GC/β-CD–rGO electrode response to pH (A), incubation time (B), β-CD–rGO suspension concentration (C) and β-CD/GO mass ratio (D) obtained from Taguchi design.

Taguchi design method was employed to study the effects of electrolyte pH and incubation time of CM on the modified electrode, as well as the effect of β-CD/GO mass ratio and β-CD–rGO quantity on the GC/β-CD–rGO electrode response. This method maintains the interactions among experimental parameters. Table SI-2† shows the orthogonal array of experimental runs and their response factors according to experimental runs. Based on these results, corresponding differential pulse voltammograms of 18 experimental runs were recorded (data not shown). The main aim of experimental design is to optimize important control factors, including the CM characterization. The graphs in Fig. 8 were used to determine the optimum parameters for CM characterization. The optimum values of pH, incubation time, GO/β-CD mass ratio and β-CD–rGO concentration were found 7, 60 minutes, 0.1 and 4 mg mL^−1^, respectively. At these optimum conditions, the best response (160 μA) for CM determination was achieved. The optimum conditions from the Taguchi design are in good agreement with the data obtained by individual investigation of each parameter.

### 3.7 Detection of curcumin

**Fig. 9:**
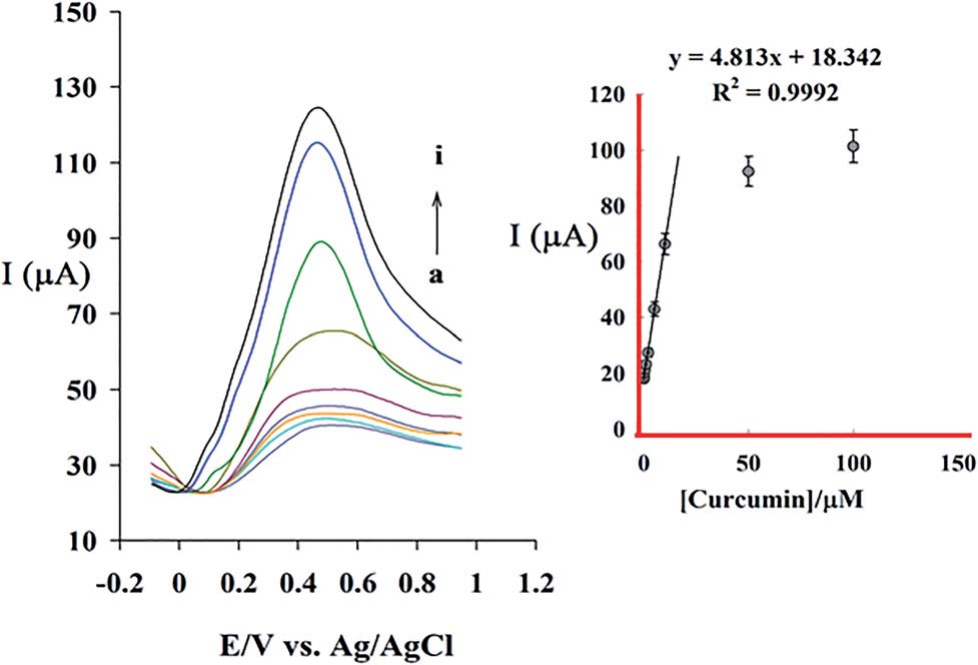
Differential pulse voltammograms of GC/β-CD–rGO electrode under optimum conditions in 0.1 M phosphate buffer solution (pH 7.0), free of CM, at the scan rate of 10 mV s^−1^, and after incubation at various CM concentrations of 0.05 (a), 0.1 (b), 0.5 (c), 1 (d), 2 (e), 5 (f), 10 (g), 100 (h) and 200 μM (i).

The relationship between oxidation peak current and CM concentration in the specified optimum condition was examined using DPV method. Fig. 9A shows differential pulse voltammograms of GC/β-CD–rGO after incubated at different concentrations of CM in phosphate buffer electrolyte solutions, free of CM. Fig. 9B shows the relationship between the peak current and concentration of CM in the range of 5 × 10^−8^ M to 2 × 10^−4^ M under the optimized condition. The sensor response increases linearly by increasing the CM concentration within the range of 5 × 10^−8^ M to 1 × 10^−5^ M with the relation of *I*_p_ (μA) = 4.813 [CM](μM) + 18.342(μA), *R*^2^ = 0.9992. Based on this equation, the detection limit (signal/noise = 3) and the sensitivity of sensor for detecting CM are calculated as 33 nM and 4.813 μA μM^−1^, respectively. Table 1 represents the comparison between β-CD–rGO sensors typical electrochemical ones for detecting CM concentration.

**Table 1:** Performance of various electrochemical curcumin sensors with different modified electrodes

Modified electrode	Method	Linear range (mol L^−1^)	Detection limit (mol L^−1^)	pH	Ref.
GCE^a^	CV^b^	9.9 × 10^−6^ to 1.7 × 10^−4^	4.1 × 10^−6^	7	56
NiCl_2_/GCES	DPV^c^	1 × 10^−5^ to 6 × 10^−4^	0.109 × 10^−6^	7	55
Poly-AVBK/GO^d^	DPV	1 × 10^−7^ to 7 × 10^−5^	4.1 × 10^−8^	6.4	57
CNT/GC^e^	FFTSWV^f^	2 × 10^−9^ to 1 × 10^−6^	5 × 10^−9^	4	58
NSrGO/Ru@AuNPs	SWV^g^	1 × 10^−10^ to 1 × 10^−12^	2 × 10^−13^	5	59
GR/GCE^h^	LSV^i^	5.0 × 10^−8^ to 3.0 × 10^−6^	3.0 × 10^−8^	1	60
ERGO/GCE^j^	CV	0.2–60.0 μM	1 × 10^−7^	7.4	49
β-CD/rGO/GC	DPV	5 × 10^−8^ to 1 × 10^−5^	33 × 10^−9^	7	This study

a Glassy carbon electrode.

b Cyclic voltammetry.

c Differential pulse voltammetry.

d Poly-AVBK graphene oxide.

e Carbon nanotube glassy carbon electrode.

f Flow injection electrochemical fast-Fourier transform square wave voltammetry.

g Square wave voltammetry.

h Graphen glassy carbon electrode.

i Linear sweep voltammetry.

j Electrochemical reduced graphene oxide glassy carbon electrode.

### 3.8 Selectivity of sensor

**Fig. 10:**
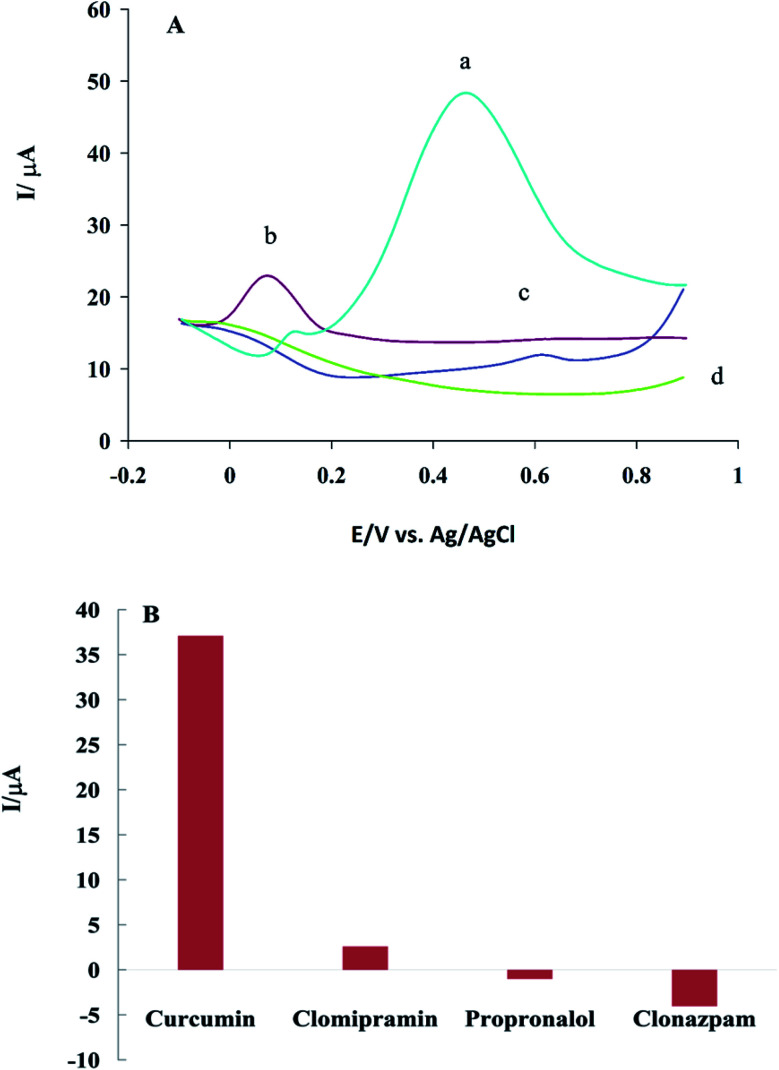
(A) Differential pulse voltammograms of GC/β-CD–rGO electrode after incubation with 1 mM CM (a), clomipramine (b), propranolol (c), and clonazepam (d). Voltammograms were recorded under optimum conditions in 0.1 M phosphate buffer solution (pH 7.0), free of CM, at the scan rate of 10 mV s^−1^. (B) The net response of GC/β-CD–rGO electrode against analyte type at the constant potential of 0.5 V.

Selectivity is one of the most important features of sensors. The selectivity of β-CD/rGO to CM was investigated with the presence of some antidepressant drugs, such as propranolol, clomipramine and clonazepam, which have polycyclic structure similar to CM. For DPV experiments, 0.1 mM of each compounds was dissolved in DMSO, and GC/β-CD–rGO electrode was immersed in it for 45 minutes, then, washed with distilled water. Fig. 10A shows the differential pulse voltammograms of GC/β-CD–rGO electrode after incubation with these drugs, under the optimum condition, in 0.1 M phosphate buffer solution free of these compounds at the scan rate of 10 mV s^−1^. Fig. 10B shows the plot of net response of GC/β-CD–rGO electrode against each drug, at a constant potential 0.5 V *vs.* Ag/AgCl electrode. As seen, these species caused no remarkable interference for characterizing CM. Thus, β-CD–rGO sensor can selectively and sensitively detect CM without any remarkable interference.

## 4 Conclusion

Herein, a simple, affordable, sensitive and selective CM electrochemical sensor was fabricated based on β-CD–rGO modified GC electrode. CM can form inclusion complex with β-CD molecules in β-CD–rGO nanocomposite while graphene nanosheets can accelerate electron transfer at the surface of modified electrode. Experimental results showed that β-CD–rGO nanocomposite greatly increased the electron transfer kinetics and provided a suitable environment for constructing modified electrodes for sensory and biosensory applications. The mass ratio β-CD/rGO and the thickness of β-CD–rGO layer had great effects on the electrochemical characterization of CM. Experimental data and Taguchi method were used to find the optimized pH, incubation time, β-CD/rGO mass ratio and the concentration of electrode modifier. Good agreement was obtained between experimental data and the results from Taguchi experimental design. These findings revealed that the optimized pH, incubation time, β-CD/rGO mass ratio and the concentration of electrode modifier were independent in the sensor fabrication. Under the obtained optimum conditions (7, 60 minutes, 0.1 and 4 mg mL^−1^ for, respectively, pH, preconcentration time, β-CD/rGO mass ratio and β-CD–rGO concentration), the fabricated electrochemical sensor had a very good analytical performance in comparison with similar electrochemical CM sensors. The fabricated sensor has a potential for various electrochemical sensors.

## List of abbreviations

β-CD: Beta-cyclodextrin
GO: Graphene oxide
rGO: Reduced grapheme oxide
CM: Curcumin
NF-κB: Nuclear factor kappa B
COX2: Cyclooxygenase 2
TNF-α: Tumour necrosis factor-α
DMSO: Dimethyl sulfoxide
GC: Glassy carbon
FTIR: Fourier transform infrared
EIS: Electrochemical impedance spectroscopy
DPV: Differential pulse voltammetry
HRTEM: High-resolution transmission electron microscopy
GCE: Glassy carbon electrode
CV: Cyclic voltammetry
Poly-AVBK/GO: Poly-AVBK graphene oxide
CNT/GC: Carbo nanotube glassy carbon electrode
FFTSWV: Flow injection electrochemical fast-Fourier transform square wave voltammetry
SWV: Square wave voltammetry
GR/GCE: Graphene glassy carbon electrode
LSV: Linear sweep voltammetry
ERGO/GCE: Electrochemical reduced graphene oxide glassy carbon electrode

## Conflicts of interest

The authors declare no conflict of interest.

## Supplementary Material

RA-011-D0RA10701H-s001
