# Supplementary material for: A reduced graphene oxide-β-cyclodextrin nanocomposite-based electrode for electrochemical detection of curcumin
Source: RSC Adv. 2021 Feb 18;11(14):7862–72. doi: 10.1039/d0ra10701h (PMC8695096; doi:10.1039/d0ra10701h)
Supplement: RA-011-D0RA10701H-s001 [file RA-011-D0RA10701H-s001.pdf]

Electronic Supplementary Material (ESI) for RSC Advances.
This journal is © The Royal Society of Chemistry 2021

Reduced Graphene Oxide-β-Cyclodextrin Nanocomposite-based Electrode for Electrochemical detection of Curcumin

Behzad Mirzaei[a], Ali Zarrabi[a,b*], Abdollah Noorbakhsh[c], Abbas Amini[d,e], Pooyan Makvandi[f,*]

[a] Department of Biotechnology, Faculty of Advanced Sciences and Technologies, University of Isfahan, Isfahan, Iran

[b] Sabanci University, Nanotechnology Research and Application Center (SUNUM), Tuzla, 34956 Istanbul, Turkey

[c] Department of Nanotechnology Engineering, Faculty of Advanced Sciences and Technologies, University of Isfahan, Isfahan, Iran

[d] Centre for Infrastructure Engineering, Western Sydney University, Penrith 2751, NSW, Australia

[e] Department of Mechanical Engineering, Australian College of Kuwait, Mishref, Kuwait

[f] Chemistry Department, Faculty of Science, Shahid Chamran University of Ahvaz, Ahvaz 6153753843, Iran

***Corresponding authors**

E-mails: alizarrabi@sabanciuniv.edu (A. Zarrabi), Pooyanmakvandi@gmail.com (P. Makvandi)

Table S-1. Factors and their level values used for Taguchi design.

parameter	Code	Level 1	Level 2	Level 3
Electrolyte pH	A	7	8	-
Accumulation time (min)	B	30	45	60
β-CD-rGO concentration ($g\ ml^{-1}$)	C	3	4	5
β-CD to GO ratio	D	0.1	0.25	0.5

Table S-2. The basic Taguchi L_{18} orthogonal arrays.

**Run**	**Control factor and levels**				**Response**
	**A**	**B**	**C**	**D**	**factor**
**1**	1	1	1	1	101
**2**	1	1	2	2	92
**3**	1	1	3	3	11
**4**	1	2	1	1	111
**5**	1	2	2	2	112
**6**	1	2	3	3	41
**7**	1	3	1	2	111
**8**	1	3	2	3	10
**9**	1	3	3	1	83
**10**	2	1	1	3	103
**11**	2	1	2	1	94
**12**	2	1	3	2	20
**13**	2	2	1	2	46
**14**	2	2	2	3	82
**15**	2	2	3	1	50
**16**	2	3	1	3	94
**17**	2	3	2	1	110
**18**	2	3	3	2	28
